# Trend of Cardio-Metabolic Risk Factors in Polycystic Ovary Syndrome: A Population-Based Prospective Cohort Study

**DOI:** 10.1371/journal.pone.0137609

**Published:** 2015-09-11

**Authors:** Fahimeh Ramezani Tehrani, Seyed Ali Montazeri, Farhad Hosseinpanah, Leila Cheraghi, Hadi Erfani, Maryam Tohidi, Fereidoun Azizi

**Affiliations:** 1 Reproductive Endocrinology Research Center, Research Institute for Endocrine Sciences, Shahid Beheshti University of Medical Sciences, Tehran, Iran; 2 Obesity Research Center, Research Institute for Endocrine Sciences, Shahid Beheshti University of Medical Sciences, Tehran, Iran; 3 Prevention of Metabolic Disorders Research Center, Research Institute for Endocrine Sciences, Shahid Beheshti University of Medical Sciences, Tehran, Iran; 4 Endocrine Research Center, Research Institute for Endocrine Sciences, Shahid Beheshti University of Medical Sciences, Tehran, Iran; 5 Student of Public Health, School of Public Health, Shahid Beheshti University of Medical Sciences, Tehran, Iran; Suzhou University, CHINA

## Abstract

**Objective:**

To see the changes of cardio-metabolic risk factors overtime in polycystic ovary syndrome vs. control women.

**Methods:**

This study was conducted on 637 participants (85 PCOS and 552 control reproductive aged, 18–45 years) of Tehran Lipid and Glucose Study (TLGS), an ongoing population-based cohort study with 12 years of follow-up. The cardiovascular risk factors of these groups were assessed in three-year intervals using standard questionnaires, history taking, anthropometric measures, and metabolic/endocrine evaluation. Generalized estimating equation was used to analyze the data.

**Results:**

Overall mean of insulin (3.55, CI: 0.66–6.45), HOMA-IR (0.63, CI: 0.08–1.18), and HOMA-β (45.90, CI: 0.86–90.93) were significantly higher in PCOS than in healthy women after adjustment for age, BMI, and baseline levels. However, the negative interaction (follow-up years × PCOS status) of PCOS and normal women converged overtime. Comparing third follow-up with first, insulin and HOMA-IR decreased 10.6% and 5%, respectively in PCOS women; and increased 6.7% and 14.6%, respectively in controls (P<0.05). The results did not show any significant result for other cardio-metabolic variables including WC, lipid profile, FPG, 2-h PG, SBP, and DBP.

**Conclusion:**

While the insulin level and insulin resistance rate were higher in reproductive aged PCOS than in healthy women, the difference of these risk factors decreased overtime. Thus, the metabolic consequences of PCOS women in later life may be lower than those initially anticipated.

## Introduction

Polycystic ovary syndrome (PCOS) is the most common endocrine disorder in women of childbearing age, with a reported prevalence of 4–15% depending on the diagnostic criteria [1]. The primary pathophysiological defect in PCOS is controversial; it might result from a combination of androgen excess and chronic anovulation. The affected women might encounter increased risk of cardio-vascular diseases (CVD), [2,3].

Increased cardio-metabolic risk factors—which are independent of, but exacerbated by obesity—are seen in PCOS women more than in normal controls [4,5]. However, the literature regarding CVD outcomes, e.g. coronary heart disease, stroke, etc., are inconsistent and there are still uncertainties over differences in CVD related mortalities among PCOS and healthy women [6–9]. For instance, some studies revealed an increased risk for future CVD [8,10], but some others conclude the opposite [11]. Having said that CVD outcomes were not so deteriorating, as we could expect from higher cardio-metabolic risk factors in PCOS patients in the literature, we should seek the trends of these risk factors in PCOS and normal women.

However, most of current evidence usually: derived from clinical-based studies [12] with small sample sizes [8], lacked appropriate control groups [13–15], used heterogeneous diagnostic criteria [16], and did not adjust potential confounders [16]. Also, the few long-term retrospective cohort studies [2] biased by comparing past-diagnosed PCOS patients with present-recruited control groups [14]. In addition, few studies with longitudinal multiple measurements of the risk factors [4], challenge the comparison of the risk factors’ trends among PCOS and healthy subjects.

Taken together, considering the scarcity of population-based cohort studies with repeated measurements of risk factors, we aimed to investigate the trends of these risk factors in Tehranian PCOS and healthy women in a 12-year prospective population-based cohort study with approximately three-year follow-up intervals.

## Materials and Methods

### Subjects

Study subjects, all from Iranian population, were randomly selected from the Tehran Lipid and Glucose Study [17]. The TLGS, an ongoing prospective cohort, has two major components: a cross-sectional study of non-communicable diseases and associated risk factors (phase 1, 1999–2001), and a prospective follow-up study at 3-year intervals (phase 2: 2002–2005, phase 3:2006–2008, and phase 4: 2009–2011). In the TLGS, 15005 people, aged ≥3 years were invited to participate. Information on various risk factors for non-communicable diseases, demographic variables, and reproductive histories was collected during face-to-face interviews, conducted every 3 years by trained interviewers.

**Fig 1:**
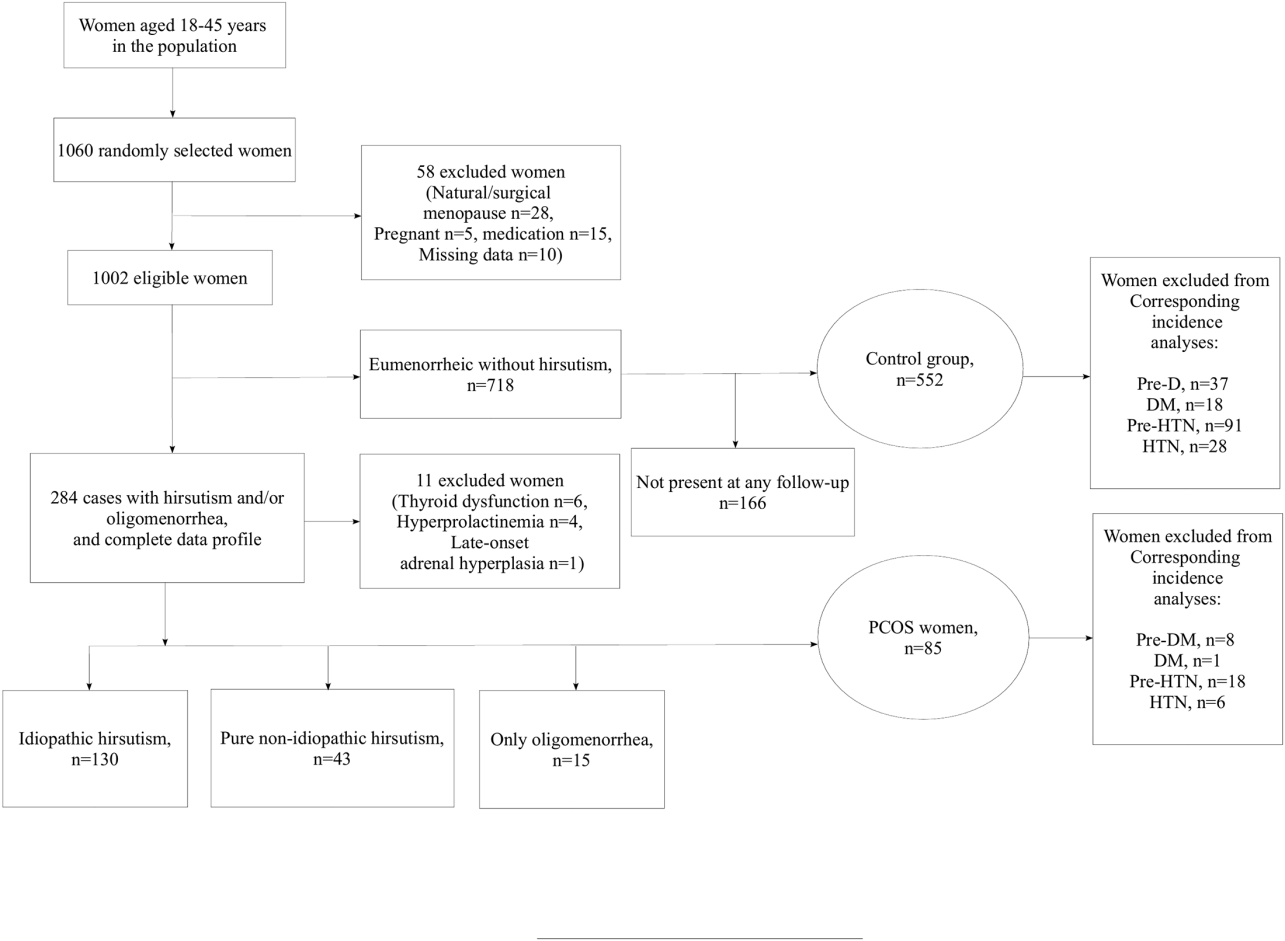
Overview of the study. (Note: The number of cases and controls diagnosed with pre-DM, DM, pre-HTN, and HTN were excluded in the corresponding analyses to report incidence rates of each disorder.)

The follow-ups included a comprehensive questionnaire, general physical examination, height and weight measurements, and taking samples of blood. For present study, of women aged 18–45 in phase 1, 1060 women were selected using systematic random sampling method and further assessed for PCOS criteria, the details of which have been published before [18]. Those women who had undergone hysterectomy or bilateral oophorectomy and those who were menopausal or pregnant, were excluded (n = 58). Information on menstrual dates and regularity, hirsutism, acne and reproductive history was collected for the 1002 eligible women using a standardized questionnaire (Fig 1). Hirsutism was assessed with the modified Ferriman-Gallwey (mFG) scoring method [19] by a trained general practitioner. Those who were on hormonal therapy were questioned about their menstrual cycle irregularity before they started medications. Patients with acne and/or first mFG score more than 3 and/or menstrual dysfunction (i.e. episodes of vaginal bleeding at 35-day intervals or more) were referred to a single endocrinologist to be re-evaluated.

Baseline blood samples, after an overnight fast, were collected between days 3 and 7 of the spontaneous menstrual cycle or progesterone-induced menstrual bleeding. A comprehensive hormonal profile testing was conducted for all study participants to further identify ovulatory dysfunction or hyperandrogenemia. In women with only hirsutism, serum levels of progesterone were measured on days 22–24 of the normal menstrual cycle to confirm ovulatory function. Except for hormonal profile, which was measured only at baseline, all other biochemical measurements were conducted at the time of recruitment and again at follow-ups, conducted every 3 years (Additional information about participant at each phases is available on S1 Table).

### Ethics Statement

The Medical Ethics Committee of the Research Institute for Endocrine Sciences approved the study protocol and the informed written consents, which were obtained from all adult participants and parents or caretakers on behalf of children or minors, were taken.

### Measurements

Subjects’ weight was measured when they were minimal clothed using a digital scale (Seca 707, Hanover, Md., USA), and rounded to the nearest 100 grams. Similarly, height was measured without shoes in standing position and normal posture of shoulders with a tape measure. Waist circumference (WC) was measured with an unstretched tape meter, at the level of umbilicus, without any pressure to body surface and recorded to the nearest 0.1 cm. Body Mass Index (BMI) was calculated as weight in kilograms (kg) divided by height squared (m^2^). Systolic (SBP) and diastolic blood pressure (DBP) were measured twice on the right arm in a seating position (by a qualified physician with a standard mercury sphygmomanometer), after the subjects sat for 15 minutes; the mean of these two measurements was considered as the subject’s SBP/DBP.

Fasting plasma glucose (FPG) was measured using the glucose oxidase method (Glucose kit; Pars Azmun, Tehran, Iran) while both the inter- and intra-assay coefficients of variation (CV) were 2.2%. The standard 2-hour plasma glucose (2-h PG, with 75g oral glucose) test was taken for those not taking glucose-lowering medications. Triglyceride (TG) levels were measured using the enzymatic colorimetric method with glycerol phosphate oxidase (TG kit; Pars Azmun, Tehran, Iran). Inter- and intra-assay CV, for TG were 0.6 and 1.6%, respectively. Total cholesterol (TC) level was determined using enzymatic colorimetric method with cholesterol esterase and cholesterol oxidase. Inter- and intra-assay CV were 0.5 and 2%, respectively. The level of high-density lipoprotein cholesterol (HDL-c) was determined after precipitation of apolipoprotein β with phosphotungstic acid and enzymatic colorimetric method (HDL-c kit; Pars Azmun, Tehran, Iran), while method sensitivity and CV were 1 mg/dl and 2.1%, respectively. Low-density lipoprotein cholesterol (LDL-c) level was calculated using the Friedewald formula [20]. Serum insulin concentration was measured using the ultrasensitive enzyme-linked radioimmunoassay method (Mercodia, Uppsala, Sweden) with a covariance <4%. Circulating levels of total testosterone (tT), androstenedione (A4), and dehydroepiandrosterone sulfate (DHEAS) were determined by Enzyme Immuno Assay method (EIA kit, Diagnostic Biochem Canada Inc.). The assay sensitivities were 0.076, 0.174, and 0.031 nmol/l, respectively; and the assay intra-assay coefficients of variation were 7.6%, 6.7%, and 5.8%, respectively. Sex hormone-binding protein (SHBG) was measured by Imuno-Enzymo Metric Assay method (IEMA kit, Diagnostic Biochem Canada Inc., Ontario, Canada). The assay sensitivity and intra-assay coefficients of variation were 0.1 nmol/l and 7.9%, respectively. The free androgen index (FAI) was calculated using the formula [tT (nmol/l) × 100/SHBG (nmol/l)].

### Definitions

Using the National Institute of Health criteria, we defined PCOS as the presence of ovulatory dysfunction and clinical hyperandrogenism and/or hyperandrogenemia, after exclusion of other known related disorders such as hyperprolactinemia and thyroid or adrenal disorders. Ovulatory dysfunction was defined as a history of eight or fewer menstrual cycles in a year, menstrual cycles of <21 or >40 days in length, or mid luteal serum progesterone levels (cycle days 22–24) of <5 ng/ml in subjects with normal regular menstrual cycle [1]. The presence of hirsutism defined as mF-G score ≥8 and was considered as clinical hyperandrogenism. Hyperandrogenemia was defined as testosterone, androstenedione or dehydroepiandrosterone sulfate levels above the 95th percentile, calculated from selected healthy non-hirsute eumenorrheic women in our study population. The details of definition of each criteria have been explained before [18]. Subjects without hirsutism or ovulatory dysfunction by history, physical examination, and hormonal profile formed our eumenorrheic non-hirsute controls. Menopause was defined, based on World Health Organization, as “permanent cessation of menstruation resulting from the loss of ovarian follicular activity.” It occurs after absence of spontaneous menstruation for 12 month or more, without any other contributable pathological or physiological cause [21].

Based on the Joint Interim Statement [22], Metabolic syndrome (MetS) was defined as presence of three or more of these criteria: FPG level ≥100 mg/dl (5.6 mmol/l) or a previously diagnosed DM; TG level ≥150 mg/dl (1.7 mmol/l) or being on treatment medications; HDL-c level <50 mg/ dl (1.3 mmol/l) or the use of cholesterol-lowering drugs; SBP ≥130 mmHg and/or DBP ≥85 mmHg or use of medications, and a waist circumference (WC) >91 cm [23].

Insulin resistance (IR) was estimated by the homeostasis model assessment (HOMA) from this formula [24]: HOMA-IR = (Fasting insulin level (mIU/l) × FPG (mmol/l)/ 22.5; a cut-off value of 2.6 for insulin resistance was considered according to the 95th percentile of HOMA-IR of 129 study participants with BMI < 25 kg/m, non-diabetic (FPG< 126 mg/dl) and non-hypertensive (SBP ≤ 130 mmHg, DBP ≤ 85 mmHg) [25]. Percent β-cell function was calculated using homeostasis model assessment: HOMA- %β = [fasting insulin level (mU/l) × 20 / (FPG (mmol/l) − 3.5)].

Definition of DM was based on the American Diabetes Association criteria [26] as FPG≥126 mg/dl (7.0 mmol/l) or 2-hPG≥200 mg/dl (11.1 mmol/l) or using medications for a previous diagnosis of DM. Pre-diabetes refers to those with impaired fasting glucose (IFG), i.e. FPG levels 100 to 125 mg/dl (5.6–6.9 mmol/l) or impaired glucose tolerance (IGT) with 2-h PG values in the oral glucose tolerance test (OGTT) of 140 mg/dl (7.8 mmol/l) to 199 mg/dl (11.0 mmol/l).

Hypertension (HTN) was defined, according to the Seventh Report of the Joint National Committee on Prevention, Detection, Evaluation, and Treatment of High Blood Pressure (JNC 7) criteria [27], as mean SBP 140 ≥mm Hg or mean DBP≥ 90 mm Hg, or current treatment for hypertension. Pre-HTN was defined by either SBP of 120–139 or DBP of 80–89.

### Statistical analyses

Continuous variables were checked for normality using the Kolmogorov–Smirnoff test. Logarithmic transformation (ln) was performed to normalize the distribution of TG, insulin, HOMA-IR, and HOMA-%β. Geometric means (CI) are presented for these values, and all other continuous data are expressed as mean ± SD.

Characteristics of women at the time of recruitment were compared between the PCOS patients and non-PCOS control group using two independent-samples t-test and analysis of covariance (ANCOVA). For categorical variables, Fisher’s exact test and logistic regression were used. The net changes of cardio-metabolic risk factors per year were calculated from the initial measurement and the last follow-up; these amounts were compared between the PCOS patients and non-PCOS group using ANCOVA (adjusted for age and baseline status of risk factor).

To analyze the incidence rates of each cardio-metabolic disorder, all patients with that disorder at the initiation of the study (phase 1) had been excluded. The incidence rates were calculated using the formula:

$$\frac{number\;of\;new\;events\;of\;the\;condition\;(cases)in\;the\;study\;time}{sum\;of\;person-time\;(person\times year)\;at\;risk\;in\;the\;study\;participants}$$

Calculation of net change per year was as: subtraction of measures of the last and first visit divided by the number of the interval years. This statistics allows us to compare the change of the variables in all subjects across the period, regardless of number of follow-ups.

Working with longitudinal data has its own pros and cons. The welcomed aspect is that they generally provide more reliable outcomes than what other observational designs like case-controls do. Additionally, the more complete data of each subject at every time points of the study, the more accurate the results. In complete datasets, the repeated measurement methods, such as multivariate analysis of variance (MANOVA), could be used. However, missing data are almost always impossible to prevent especially in cohorts. And, replacing the missing data is not the only solution. In order to overcome the problem, one can use other statistical methods as we used generalized estimating equation (GEE) in the present study. Interestingly, the results of GEE analysis on a dataset with missing data and continuous outcome variables are comparable to that obtained from a complete dataset [28]. The GEE approach, which was developed by Liang and Zeger, considers the correlation between repeated measurements and can be used in different statistical methods, including linear and logistic regression. Thus, the GEE approach is suitable for both continuous and dichotomous outcome variables [29]. The GEE analysis was performed on data of the subjects from phase 1, who had the information required in at least one of four study phases, with following predictors: Time (follow-up years), PCOS status, and an interaction term of these two (Follow-up years × Study group). This model was adjusted for age, BMI, and baseline status of each cardio-metabolic parameter.

Statistical analysis was performed using SPSS (version 16;SPSS Inc., Chicago, IL, USA) and software package STATA (version 12;STATA Inc., College Station, TX, USA); considering significance level at P< 0.05, and CI as 95%.

## Results

Of 1002 women recruited from TLGS [18], 637 participants including 85 (13.3%) with PCOS diagnosis and 552 (85.7%) as eumenorrheic non-hirsute controls were entered the study. Of these participants, 375 (58.9%, 51 PCOS and 324 controls) were present at all three follow-ups, 155 (24.3%, 24 PCOS and 131 controls) missed just one follow-up and 107 (16.8%, 10 PCOS and 97 controls) missed two. Consequently, all patients in this study had at least one follow-up (Fig 1). The median and inter-quartile range for follow-up years of PCOS and healthy women were 9.4 (8.7–10.4) and 9.5 (8.7–10.6), respectively.

PCOS women had significantly higher BMI, WC, TC, and MetS prevalence than non-PCOS controls at the initiation of the study; these differences disappeared after adjustment for age and BMI. No significant difference was seen in the prevalence of pre-DM, DM, pre-HTN, and HTN between PCOS and healthy women at the initial phase; the prevalence of pre-diabetes in PCOS patients and non-PCOS women at the time of recruitment were 11.1% (95% CI: 4.9%-20.7%) and 7.3% (95% CI: 5.2%-9.9%), respectively. Diabetes’ prevalences were 1.2% (95% CI: 0.0%-6.6%) in PCOS patients and 3.3% (95% CI: 2.0%-5.2%) in non-PCOS subjects. The prevalence of pre-hypertension were 26.5% (95% CI: 16.5%-38.6%) and 19.7% (95% CI: 16.1%-23.6%), and of hypertension were 7.1% (95% CI: 2.7%-14.9%) and 5.1% (95% CI: 3.4%-7.3%), in PCOS patients and non-PCOS subjects, respectively (Table 1).

**Table 1:** Baseline Characteristics of Study participants (PCOS and control women).

	PCOS	Normal
**Number of Subjects**	**85**	**552**
**Marital status** ^c^		
Single	20 (27%)	157 (28.4%)
Married	51 (68.9%)	383 (69.4%)
Divorced/widowed	3 (4.1%)	12 (2.2%)
**Education level** ^c^		
Illiterate	1 (1.4%)	8 (1.4%)
Primary	31 (41.9%)	244 (44.2%)
Secondary	34 (45.9%)	257 (46.6%)
High	8 (10.8%)	43 (7.8%)
**Parity** ^c^	2.5±1.6	2.4±1.2
**Number of children**	2.4±1.5	2.3±1.2
**Menopause**	0	0
**Smoking** ^c^	1.4%	2.4%
**PA** ^c^		
Low	64.9%	60.6%
Moderate	13.5%	18%
High	21.6%	21.4%
**FAI** ^a^	**3.3±1.7** ^d^	**2.5±1.4** ^d^
**tT** ^a^ (nmol/l)	**0.6±0.5** ^e^	**0.4±0.2** ^e^
**SHBG** ^a^ (nmol/l)	**36.9±11.4** ^d^ ^,^ ^e^	**47.9±10.9** ^d^ ^,^ ^e^
**DHEAS** ^a^ (nmol/l)	114.1±64.3	105.9±49.1
**A4** ^b^ (nmol/l)	**1.2±2.8** ^d^	**0.7±1.6** ^d^
**Age** ^a^ (years)	29.8±9.2	29.3±9.0
**BMI** ^a^ (kg/m^2^)	**27.2±5.3** ^d^	**25.6±5.0** ^d^
**WC** ^a^ (cm)	**86.1±13.5** ^d^	**83.0±12.2** ^d^
**TC** ^a^ (mmol/l)	**5.1±1.1** ^d^	**4.9±1.0** ^d^
**LDL-c** ^a^ (mmol/l)	3.2±0.9	3.1±0.9
**HDL-c** ^a^ (mmol/l)	1.2±0.3	1.2±0.3
**TG** ^a^ (mmol/dl)	**1.4±0.8** ^d^	**1.2±0.6** ^d^
**FPG** ^a^ (mmol/l)	4.9±0.5	4.9±1.2
**2-h PG** ^a^ (mmol/l)	6.1±1.7	5.9±2.0
**Insulin** (mIU/l) ^b^	9.6±5.2	8.2±4.8
**HOMA-IR** ^b^	2.1±1.2	1.8±1.1
**IR** ^c^	29.3%	21.6%
**HOMA-%β** ^b^	147.1±83.9	123.8±80.0
**SBP** ^a^ (mmHg)	109.9±11.2	108.5±12.1
**DBP** ^a^ (mmHg)	73.8±9.8	72.3±9.3
**Pre DM** ^c^	11.1%	7.3%
**DM** ^c^	1.2%	3.3%
**Pre HTN** ^c^	26.5%	19.7%
**HTN** ^c^	7.1%	5.1%
**MetS** ^c^	**24.1%** ^d^	**14.5%** ^d^

PCOS, polycystic ovary syndrome, based on NIH criteria

PA, physical activity; FAI, free androgen index; tT, total testosterone; SHBG, sex hormone binding globulin; DHEAS, dehydroepiandrosterone sulfate; A4, androstenedione.

BMI, body mass index; WC, waist circumference; TC, total cholesterol; LDL-c, low density lipoprotein cholesterol; HDL-c, high density lipoprotein cholesterol; TG, triglycerides; FPG, fasting plasma glucose; 2-h PG, 2-hour glucose; HOMA-IR, insulin resistance calculated by homeostasis model assessment from this formula: (fasting insulin level (mIU/l) × FPG (mmol/l)/ 22.5.

IR, insulin resistance; cut-off point for IR = 2.6 (from above formula).

HOMA-%β, homeostasis model assessment value for percent β-cell function: [fasting insulin level (mIU/l) × 20 / (FPG (mmol/l) − 3.5)].

SBP, systolic blood pressure; DBP, diastolic blood pressure; DM, diabetes mellitus; HTN, hypertension; MetS, metabolic syndrome

^a^ Data are shown as mean±SD.

^b^ Values are expressed as of geometric mean ± SD; measures were analyzed using logarithmic transformation (Ln).

^c^ Data shown as percentage.

^d^ Significant difference (P<0.05), analyzed using independent t-test for superscripts ^a, b^ and Fisher’s Exact test for superscript ^c^.

^e^ No significant difference was seen in all parameters between 2 groups after adjustment for age and BMI (Analysis of Covariance for superscripts ^a, b^, and Logistic Regression for superscript ^c^), except for tT and SHBG (P<0.05).

The mean changes of each metabolic feature are shown in Table 2, before and after splitting according to BMI status. Except for HDL-c and TG, no significant difference existed between the mean changes of metabolic parameters between PCOS patients and non-PCOS subjects. In normal BMI subgroup, increase of HDL-c per year was significantly higher among PCOS women compared with healthy controls (p-value<0.05). While TG was increased in high BMI subgroup of PCOS women, it decreased over time in both subgroups of non-PCOS women.

**Table 2:** Changes of Metabolic Parameters Per Year in PCOS and Non-PCOS Subjects.

	Total		High BMI		Normal BMI	
	PCOS	Non-PCOS	PCOS	Non-PCOS	PCOS	Non-PCOS
**BMI** (kg/m^2^) ^a^	0.21±0.35	0.26±0.37	0.15±0.37	0.16±0.40	0.30±0.30	0.35±0.30
**WC** (cm) ^a^	0.68±1.16	0.72±1.13	0.38±1.09	0.55±1.17	1.15±1.12	0.88±1.06
**TC** (mmol/l) ^a^	-0.03±0.11	-0.01±0.10	-0.04±0.12	-0.03±0.11	-0.00±0.09	0.01±0.08
**LDL-c** (mmol/l) ^a^	-0.05±0.08	-0.03±0.09	-0.06±0.09	-0.04±0.10	-0.02±0.07	-0.01±0.07
**HDL-c** (mmol/l) ^a^	0.01±0.03	0.01±0.03	0.01±0.03	0.01±0.03	0.02±0.03 ^c^	0.01±0.03 ^c^
**TG** (mmol/l)^b^	1.00±0.07	1.00±0.06	1.01±0.08 ^c^	0.99±0.05 ^c^	1.00±0.04	1.01±0.06
**SBP** (mmHg) ^a^	0.82±4.09	0.82±3.95	0.23±4.08	1.04±4.47	1.74±3.72	0.53±3.67
**DBP** (mmHg) ^a^	0.05±1.42	0.06±1.27	0.04±1.58	0.05±1.32	0.02±1.13	0.04±1.22
**FPG** (mmol/l) ^a^	0.04±0.11	0.03±0.12	0.03±0.12	0.03±0.14	0.03±0.06	0.03±0.10
**Insulin** (mIU/l) ^b^	1.00±0.06	1.00±0.06	1.01±0.05	1.00±0.05	0.97±0.06	1.00±0.07
**HOMA-IR** ^b^	1.01±0.06	1.01±0.06	1.01±0.06	1.01±0.06	0.98±0.06	1.01±0.07
**HOMA-%β** ^b^	0.96±0.05	0.97±0.06	0.98±0.05	0.97±0.06	0.93±0.05	0.98±0.07

PCOS, polycystic ovary syndrome, based on NIH criteria;

BMI, body mass index; WC, waist circumference; TC, total cholesterol; LDL-c, low density lipoprotein cholesterol; HDL-c, high density lipoprotein cholesterol; TG, triglycerides; FPG, fasting plasma glucose; SBP, systolic blood pressure; DBP, diastolic blood pressure;

HOMA-IR, homeostasis model assessment value for insulin resistance: [fasting insulin level (mIU/l) × FPG (mmol/l)/ 22.5]; HOMA-%β, homeostasis model assessment value for percent β-cell function: [fasting insulin level (mIU/l) × 20 / (FPG (mmol/l) − 3.5)].

High BMI, BMI≥25 (kg/m^2^); Normal BMI, BMI<25 (kg/m^2^)

^a^ Values are expressed as changes of mean ± SD.

^b^ Values are expressed as changes of geometric mean ± SD.

^c^ Significant difference (p-value < 0.05) after adjustment for age and baseline status

Re-analyzing the patients, who were taking medications, did not change the results. Also, when we analyzed only those presenting at all follow-ups, the results did not change significantly.

**Fig 2:**
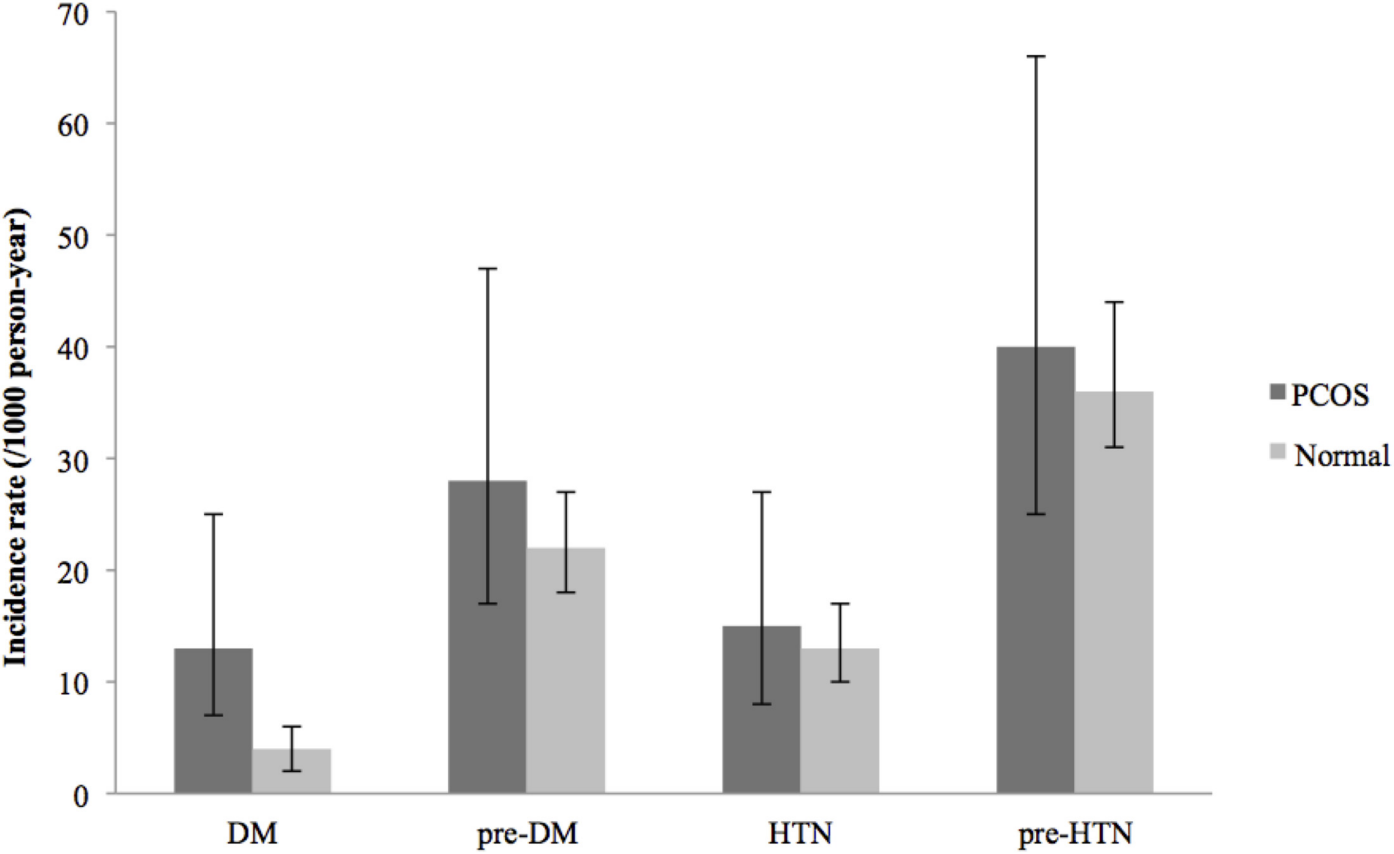
Incidence rates (/1000 person-year) of pre-DM (pre-diabetes), DM (diabetes), pre-HTN (pre-hypertension), and HTN (hypertension). The bar chart and error bars show mean and 95% confidence intervals, respectively.

During the study, incidence rates of DM in PCOS women and non-PCOS controls differed significantly: 13(95% CI: 7–25) versus 4(95% CI: 2–6)/1000 person-years, respectively. However, no significant difference was observed in the incidence of pre-DM, pre-HTN, or HTN (Fig 2).

**Fig 3:**
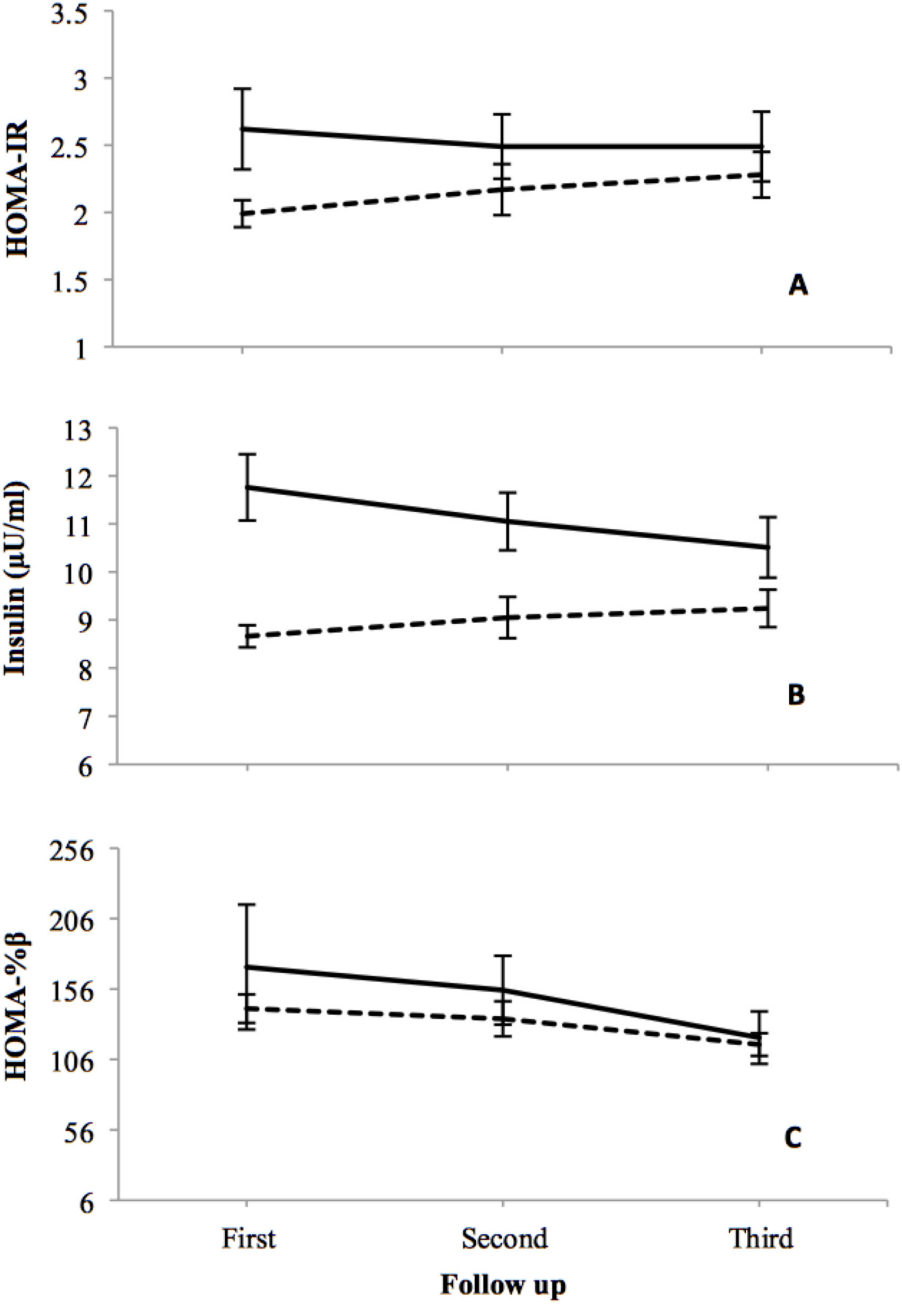
GEE estimated measures of HOMA-IR (A), insulin (B), and HOMA-%β (C) in PCOS (dark line) and non-PCOS (dashed line) women at 3 follow-ups assuming the interaction between time and study group and also adjusting for age, BMI, and baseline insulin. The patterns of mean changes are different in the PCOS subjects and controls. Error bars indicate standard deviation.

Based on GEE analysis, overall mean of insulin, HOMA-%β and HOMA-IR were significantly higher (p<0.05) in PCOS than in healthy women, after adjustment for age, BMI, and baseline status of these variables. However, the negative interaction (follow-up years × PCOS status) in PCOS and non-PCOS women converged over time (Fig 3). This means that if the groups were considered to have the same insulin and/or HOMA-IR at baseline, they develop differently over time having the same age and BMI (Table 3). Comparison of the third follow-up (phase 4) with the baseline visit (phase 1) demonstrated that insulin level and HOMA-IR of PCOS patients decreased by 10.6% and 5%, respectively. Nevertheless, these values in healthy subjects increased 6.7% and 14.6%, respectively.

**Table 3:** Parameter Estimates of GEE Model in PCOS Compared with Non-PCOS Women.

Dependent Variable	Parameter	Beta	Standard Error	95% Wald Confidence Interval	*P*-Value
**Insulin**					
	**PCOS**	3.55	1.48	(0.66, 6.45)	**0.016**
	**Non-PCOS**	Reference			
	**Age**	-0.06	0.03	(-0.12, -0.01)	**0.023**
	**BMI**	0.33	0.06	(0.22, 0.44)	**<0.001**
	**Baseline Insulin**	0.12	0.02	(0.08, 0.16)	**<0.001**
	**Time**	0.07	0.05	(-0.03, 0.17)	0.156
	**PCOS** * **Time**	-0.31	0.14	(-0.58, -0.03)	**0.028**
	**Non-PCOS** * **Time**	Reference			
**HOMA-IR**					
	**PCOS**	0.63	0.28	(0.08, 1.18)	**0.025**
	**Non-PCOS**	Reference			
	**Age**	0.00	0.01	(-0.01, 0.01)	0.940
	**BMI**	0.06	0.02	(0.02, 0.11)	**0.007**
	**Baseline HOMA-IR**	0.46	0.07	(0.31, 0.60)	**<0.001**
	**Time**	0.04	0.01	(0.01, 0.066)	**0.007**
	**PCOS** * **Time**	-0.06	0.31	(-0.12, 0.00)	**0.041**
	**Non-PCOS** * **Time**	Reference			
**IR** ^a^					
	**PCOS**	3.36		(1.27, 8.89)	**0.015**
	**Non-PCOS**	Reference			
	**Age**	1.00		(0.97, 1.03)	0.919
	**BMI**	1.12		(1.06, 1.18)	**<0.001**
	**Baseline IR+**	5.02		(3.01, 8.37)	**<0.001**
	**Baseline IR-**	Reference			
	**Time**	1.13		(1.05, 1.23)	**0.001**
	**PCOS** * **Time**	0.89		(0.81, 0.98)	**0.019**
	**Non-PCOS** * **Time**	Reference			
**HOMA-%β**					
	**PCOS**	45.90	22.98	(0.86, 90.93)	**0.046**
	**Non-PCOS**	Reference			
	**Age**	-1.23	0.40	(-2.02, -0.44)	**0.002**
	**BMI**	2.09	0.71	(0.70, 3.48)	**0.003**
	**Baseline HOMA-%β**	0.31	0.06	(0.20, 0.43)	**<0.001**
	**Time**	-3.39	0.68	(-4.72, -2.06)	**<0.001**
	**PCOS** * **Time**	-4.82	2.54	(-9.80, 0.16)	0.058
	**Non-PCOS** * **Time**	Reference			

GEE, generalized estimating equation

PCOS, polycystic ovary syndrome, based on NIH criteria

BMI, body mass index

HOMA-IR, insulin resistance calculated by homeostasis model assessment from this formula: (fasting insulin level (mIU/l) × FPG (mmol/l)/ 22.5.

IR, insulin resistance; cut-off point for IR = 2.6 (from above formula)

HOMA-%β, homeostasis model assessment value for percent β-cell function: [fasting insulin level (mIU/l) × 20 / (FPG (mmol/l) − 3.5)].

^a^ Odds ratios for insulin resistance are shown in parameter column.

* Indicates interaction.

The overall chance of IR in PCOS patients was 3.4 (95%CI: 1.3–8.9) times that of the control group. However, because of the protective effect of interaction (time × study group), the odds of IR decreased by 11% (95%CI: 2%-19%) yearly in PCOS, compared with the non-PCOS women.

GEE analyses did not illustrate any significant results for other cardio-metabolic variables including WC, TC, LDL-c, HDL-c, TG, TC/HDL-c, TG/ HDL-c FPG, 2-h PG, SBP, and DBP (S2 Table).

## Discussion

To the best of our knowledge, this is the first population-based cohort study with GEE analysis which demonstrates that while the overall mean of insulin level and IR was higher in PCOS than non-PCOS women, this difference fades away overtime. In other words, in spite of the increase in insulin level and IR in healthy ones, they decrease in PCOS women. We have not found any significant differences in other cardio-metabolic risk factors between PCOS and non-PCOS women; a finding in contrary to that of some other studies [30,31].

Insulin resistance may result from several mechanisms in PCOS women: decreased insulin secretion and/or hepatic clearance, defected gluconeogenesis in the liver and impaired signaling pathways or receptors of insulin. IR could promote CVDs directly through sympathetic overactivity, endothelial function and vascular reactivity pathologies; and/or indirectly via damaging fibrinolysis, impairing lipolysis suppression, and inducing hypertension [32].

The relationship between CVD outcomes and PCOS is still the matter of debate [6]: while some studies showed significant differences in CVD outcomes between PCOS and non-PCOS women [7,10,33], others concluded no evidence of different CVD outcomes between the two groups [8,9,11]. These paradoxical observations might result from differences in study designs and characteristics of the participants such as age, BMI, reproductive status, ethnicities, and lifestyles. Moreover, clinical-based studies might be misleading; they present severe phenotypes of PCOS women referred for treatment. On the other hand, a population-based study might include younger, lower BMI women with less severe phenotypes that might have never been referred. Besides, the majority of current studies: had small sample sizes [8], lacked appropriate control groups [13–15], did not adjust strong confounders [16], did not have multiple measurements of the risk factors across time, and did not simultaneously recruit PCOS and healthy subjects [14].

The assumed paradox of early high prevalence of cardio-metabolic risk factors and no increased rate of mortality in PCOS women could be explained by several mechanisms [11,34,35]. First, assuming that CVD risks appear later than IR in the cascade of cardiovascular events, and healthy controls develop the risk factors more rapidly, one might not expect the theoretical rise of CVD morbidity and mortality in PCOS women. Second, along with our findings, the higher waist to hip ratio in PCOS women disappeared overtime mainly due to weight loss consideration in PCOS and weight gaining among non-PCOS women [36]. Third, compared with healthy controls, CVD risk factors might be more prominent in younger PCOS women than in older ones [37]. Fourth, it might be the irregular menstrual cycles that associate with increased age-adjusted risk for CVD mortality; however, this risk might attenuate after accounting for BMI [38]. Fifth, several reproductive characteristics of PCOS women may also explain that paradox such as late menarche [39], late menopause, fewer children, later pregnancy [40], extended reproductive lifespan [41] and re-establishment of the negative feedback loops in the hypothalamic-pituitary-gonadal (HPG) axis [42]. Sixth, it seems that the reproductive aging process is delayed in PCOS women, giving them a longer exposure to endogenous estrogen and the reproductive phase of HPG axis. Also, considering the PCOS phenotype as a determining factor for cardio-metabolic risk factors [43], this might improve with aging [44]. The possible cardio-protective effect of this reproductive longevity could possibly overcome the adverse effects of PCOS at younger ages. PCOS women conceive at later ages and have fewer children On the other hand, maternal mortality increases by parity, and maternal age at first pregnancy positively associates with lifespan [40]. As a result, this delayed reproduction and lower conception rate may partly explain the CVD paradox observed in PCOS.

We have also demonstrated that metabolic disturbances of PCOS women are highly influenced by obesity across time; while TG decreased in non-PCOS women (regardless of their BMI) overtime, it increased in overweight/obese subgroup of PCOS women. Besides, normal BMI subgroup of PCOS participants had a significantly higher increase per year in HDL-c level compared with healthy controls. Although the amount of this difference is slight, it can be clinically significant in a long enough follow-up. Some studies revealed no difference in cardio-metabolic risk factors between weight-matched PCOS women and healthy controls [14,45–47]; however, the majority of them showed that cardio-metabolic risk factors (e.g. dyslipidemia, hypertension, insulin resistance, pancreatic β-cell dysfunction, IGT, DM2, and MetS) are more common in PCOS than in non-PCOS women [2–5]—independent of, but exacerbated by obesity [15,16,48–50].

Our study has a number of strengths. It was a population-based prospective cohort with 12 years of follow-up and several precise measurements of cardio-metabolic risk factors. Moreover, as an ongoing study, it enables us to follow them for further cardio-metabolic events. Also, the precise measurements and statistical analysis with specific adjustments of age, BMI, and baseline status for each variable could help the study to reach more powerful results. In addition, as subjects were not selected in a clinical-based situation, the results could be more accurate, when generalizing to population. However, a potential limitation can be the fact that the development of cardiovascular risk is a major long-term event that needs long follow-ups. Also, we have used HOMA-IR as a surrogate marker for assessing of IR. In spite of good correlation between HOMA-IR and gold standard clamp methods [51], it might be inaccurate in PCOS subjects [52]. We have not had enough power to run subgroup analysis according to various PCOS phenotypes. Furthermore, we have not collected data regarding some lifestyle modifications and dietary habits. Unfortunately, we have collected hormonal data of the subjects only in the first phase and did not have these data in follow-ups. Also, Some kinds of misclassification might have happened, as we have not reevaluated the controls’ PCOS status at later phases. Finally, like many other cohort studies, some cases had missed at least one follow-up; however, as no significant difference exists between baseline characteristics of those subjects who missed the follow-ups and those who did not (data have not been shown), it may not have significantly changed our results. Considering length of follow-up and age of participants at baseline, the majority of participants still were in premenopausal state. Therefore, we could not assess the impact of menopause.

## Conclusion

Despite higher mean of insulin level and IR in reproductive-aged PCOS women than in healthy ones, these gaps converge gradually; as controls had caught up with the previous differences of risk profiles by time. However, this conclusion might be taken with caution until larger cohorts of longer follow-up periods will be done.

## Supporting Information

S1 Table Metabolic Characteristics of PCOS and Normal Subjects in Study Phases. (DOCX)

S2 Table GEE Model for metabolic parameters of PCOS and Normal participants. (DOCX)
