# Supplementary material for: Trend of Cardio-Metabolic Risk Factors in Polycystic Ovary Syndrome: A Population-Based Prospective Cohort Study
Source: PLoS One. 2015 Sep 11;10(9):e0137609. doi: 10.1371/journal.pone.0137609 (PMC4567354; doi:10.1371/journal.pone.0137609)
Supplement: S1 Table — (DOCX) [file pone.0137609.s001.docx]

| **SUPPLEMENTARY TABLE 1. Metabolic Characteristics of PCOS and Normal Subjects in Study Phases.** | | | | | | | | |
| --- | --- | --- | --- | --- | --- | --- | --- | --- |
|  | **Baseline status** | | **1^st^ follow-up** | | **2^nd^ follow-up** | | **3^rd^ follow-up** | |
|  | **PCOS** | **Normal** | **PCOS** | **Normal** | **PCOS** | **Normal** | **PCOS** | **Normal** |
| **Number of subjects** | **85** | **552** | **69** | **428** | **73** | **439** | **69** | **464** |
| **Follow-up-intervals**^a^ | NA | NA | 3.30 (2.75-4.19) | 3.69 (2.69-4.15) | 2.85 (2.30-3.69) | 2.71 (2.15-3.18) | 3.12 (2.73-3.46) | 3.21 (2.84-3.61) |
| **Age**^b^(years) | 29.78±9.21 | 29.35±8.99 | 33.46±9.60 | 33.38±8.90 | 36.47±9.27 | 36.08±8.95 | 39.86±9.53 | 39.56±8.99 |
| **BMI**^b^(kg/m^2­­^) | **27.24±5.35 ^e^** | **25.61±4.96^e^** | 28.03±5.72 | 26.90±4.92 | **28.81±5.58^e^** | **27.12±4.70^e^** | 29.40±5.73 | 28.13±4.86 |
| **WC**^b^(cm) | **86.10±13.48^e^** | **82.97±12.24^e^** | 86.90±13.47 | 84.95±12.34 | **89.07±13.40^e^** | **84.04±12.21^e^** | 92.84±11.72 | 90.55±11.54 |
| **TC**^b^ (mmol/l) | **5.14±1.14^e^** | **4.89±1.02^e^** | 4.81±1.05 | 4.61±0.91 | 4.89±1.08 | 4.69±0.89 | 4.93±0.96 | 4.82±0.94 |
| **LDL-c** ^b^ (mmol/l) | 3.22±0.93 | 3.11±0.87 | 3.03±0.89 | 2.88±0.81 | 2.91±0.82 | 2.91±0.77 | 2.87±0.78 | 2.86±0.81 |
| **HDL-c** ^b^ (mmol/l) | 1.18±0.30 | 1.16±0.27 | 1.11±0.29 | 1.07±0.27 | 1.19±0.31 | 1.17±0.26 | 1.33±0.29 | 1.31±0.30 |
| **TG** ^c^(mmol/l) | **1.38±0.83^e^** | **1.20±0.62^e^** | 1.33±0.68 | 1.26±0.63 | **1.45±0.87 ^e, f^** | **1.20±0.60 ^e, f^** | 1.39±0.89 | 1.25±0.66 |
| **FPG**^b^(mmol/l) | 4.89±0.53 | 4.92±1.17 | 4.94±0.66 | 4.96±1.26 | 5.06±1.12 | 4.98±1.27 | 5.25±0.82 | 5.30±1.36 |
| **2-h PG**^b^(mmol/l) | 6.12±1.72 | 5.94±2.01 | 6.29±2.11 | 5.86±1.95 | **5.91±1.89^e^** | **5.48±1.58^e^** | **6.27±1.76 ^d^** | **5.77±1.73 ^d^** |
| **Insulin**(mIU/l) ^c^ | 9.62±5.17 | 8.16±4.78 | 9.31±7.06 | 8.19±3.71 | 9.00±5.31 | 7.51±3.84 | 9.33±5.71 | 8.31±4.34 |
| **HOMA-IR** ^c^ | 2.08±1.18 | 1.77±1.09 | 2.04±1.63 | 1.80±0.92 | 2.00±1.39 | 1.65±0.97 | 2.20±1.53 | 1.94±1.14 |
| **IR** ^d^ | 29.3% | 21.6% | 36.1% | 21.6% | **35.9%^g^** | **18.8%^g^** | 38.1% | 30.4% |
| **HOMA-%β** ^c^ | 147.06±83.88 | 123.84±80.00 | 136.80±95.15 | 123.40±69.70 | 136.91±75.35 | 113.05±67.23 | 107.71±55.98 | 100.51±57.21 |
| **SBP**^b^(mmHg) | 109.93±11.25 | 108.47±12.10 | 105.35±10.90 | 107.18±13.78 | 107.65±14.27 | 106.19±14.33 | 109.41±14.76 | 109.36±16.29 |
| **DBP**^b^(mmHg) | 73.79±9.79 | 72.34±9.26 | 70.57±8.84 | 70.98±9.68 | 69.78±11.04 | 69.95±10.13 | 74.94±11.50 | 73.82±10.95 |
| **MetS**^d^ | **24.1%^g^** | **14.5%^g^** | 24.6% | 20.7% | 21.7% | 17.7% | 32.4% | 22.9% |
| **Menopause**^d^ | 0 | 0 | 3 (4.3%) | 3 (0.7%) | 3 (4.2) | 13 (3.1%) | 4 (6.5%) | 46 (10.3%) |
| PCOS, polycystic ovary syndrome, based on NIH criteria  BMI, body mass index; WC, waist circumference; TC, total cholesterol; LDL-c, low density lipoprotein cholesterol; HDL-c, high density lipoprotein cholesterol; TG, triglycerides; FPG, fasting plasma glucose; 2-h PG, 2-hour glucose; HOMA-IR, insulin resistance calculated by homeostasis model assessment from this formula: [fasting insulin level (mIU/l) × FPG (mmol/l)/ 22.5].  IR, insulin resistance; cut-off point for IR=2.6 (from above formula).  HOMA-%β, homeostasis model assessment value for percent β-cell function: [fasting insulin level (mIU/l) × 20 / (FPG (mmol/l) − 3.5)].  SBP, systolic blood pressure; DBP, diastolic blood pressure; MetS, metabolic syndrome  ^a^Data are shown as median and interquartile ranges (IQ 25-75).  ^b^Data shown as mean ± SD, differences between PCOS and normal subjects were analyzed using independent t-test, and Analysis of Covariance (ANCOVA), adjusted for age and BMI.  ^c^Values are expressed as of geometric mean ± SD; measures were analyzed using logarithmic transformation (Ln).  ^d^Data shown as percentage, differences between PCOS and normal subjects were analyzed using Fisher’s Exact test, and Logistic Regression, adjusted for age and BMI.  ^e^Significant difference (p-value<0.05) was seen using independent t-test.  **^f^**Significant difference were seen using ANCOVA, adjusted for age and BMI  ^g^Significant difference (p-value<0.05) was seen usingFisher’s Exact test. | | | | | | | | |
|  |  |  |  |  |  |  |  |  |
|  |  |  |  |  |  |  |  |  |
