# Supplementary material for: Trend of Cardio-Metabolic Risk Factors in Polycystic Ovary Syndrome: A Population-Based Prospective Cohort Study
Source: PLoS One. 2015 Sep 11;10(9):e0137609. doi: 10.1371/journal.pone.0137609 (PMC4567354; doi:10.1371/journal.pone.0137609)
Supplement: S2 Table — (DOCX) [file pone.0137609.s002.docx]

| **SUPPLEMENTAL TABLE 2. GEE Model for metabolic parameters of PCOS and Normal participants** | | | | | | |
| --- | --- | --- | --- | --- | --- | --- |
| **Dependent Variable** | **Parameter** | **Beta** | **Standard Error** | **95% Wald Confidence Interval** | | ***P*-Value** |
| **WC** |  |  |  |  |  |  |
|  | **PCOS** | 0.394 | 1.2865 | (-2.128, 2.916) | | 0.759 |
|  | **Normal** | Reference |  |  | |  |
|  | **Age** | 0.153 | 0.0360 | (0.082, 0.223) | | **<0.001** |
|  | **BMI** | 1.126 | 0.1060 | (0.918, 1.334) | | **<0.001** |
|  | **Baseline WC** | 0.283 | 0.0412 | (0.203, 0.364) | | **<0.001** |
|  | **Time** | 0.978 | 0.0684 | (0.844, 1.112) | | **<0.001** |
|  | **PCOS*Time** | 0.008 | 0.1823 | (-0.350, 0.365) | | 0.966 |
|  | **Normal*Time** | Reference |  |  | |  |
| **BMI** |  |  |  |  |  |  |
|  | **PCOS** | 0.093 | 0.399 | (-0.688, 0.055) | | 0.815 |
|  | **Normal** | Reference |  |  | |  |
|  | **Age** | -0.019 | 0.012 | (-0.042, 0.005) | | 0.115 |
|  | **Baseline BMI** | 0.883 | 0.024 | (0.835, 0.931) | | **<0.001** |
|  | **Time** | 0.241 | 0.019 | (0.203, 0.279) | | **<0.001** |
|  | **PCOS*Time** | -0.009 | 0.064 | (-0.134, 0.117) | | 0.892 |
|  | **Normal*Time** | Reference |  |  | |  |
| **TC** |  |  |  |  |  |  |
|  | **PCOS** | 0.100 | 0.1331 | (-0.161, 0.360) | | 0.454 |
|  | **Normal** | Reference |  |  | |  |
|  | **Age** | 0.019 | 0.003 | (0.013, 0.025) | | **<0.001** |
|  | **BMI** | -0.014 | 0.0053 | (-0.024, -0.004) | | **0.009** |
|  | **Baseline TC** | 0.579 | 0.0251 | (0.529, 0.628) | | **<0.001** |
|  | **Time** | 0.030 | 0.0060 | (0.019, 0.042) | | **<0.001** |
|  | **PCOS*Time** | -0.011 | 0.0177 | (-0.046, 0.024) | | 0.531 |
|  | **Normal*Time** | Reference |  |  | |  |
| **LDL-c** |  |  |  |  | |  |
|  | **PCOS** | 0.147 | 0.1140 | (-0.077, 0.370) | | 0.197 |
|  | **Normal** | Reference |  |  | |  |
|  | **Age** | 0.013 | 0.0025 | (0.008, 0.018) | | **<0.001** |
|  | **BMI** | -0.011 | 0.0048 | (-0.021, -0.002) | | **0.017** |
|  | **Baseline LDL-C** | 0.594 | 0.0256 | (0.544, 0.644) | | **<0.001** |
|  | **Time** | -0.005 | 0.0053 | (-0.016, 0.005) | | 0.327 |
|  | **PCOS*Time** | -0.025 | 0.0160 | (-0.056, 0.007) | | 0.121 |
|  | **Normal*Time** | Reference |  |  | |  |
| **HDL-c** |  |  |  |  | |  |
|  | **PCOS** | 0.017 | 0.0408 | (-0.063, 0.097) | | 0.670 |
|  | **Normal** | Reference |  |  | |  |
|  | **Age** | 0.000 | 0.0009 | (-0.002, 0.002) | | 0.855 |
|  | **BMI** | -0.002 | 0.0018 | (-0.005, 0.002) | | 0.285 |
|  | **Baseline HDL-C** | 0.619 | 0.0388 | (0.543, 0.695) | | **<0.001** |
|  | **Time** | 0.036 | 0.0021 | (0.032, 0.040) | | **<0.001** |
|  | **PCOS*Time** | 0.001 | 0.0052 | (-0.009, 0.011) | | 0.888 |
|  | **Normal*Time** | Reference |  |  | |  |
| **TG** |  |  |  |  | |  |
|  | **PCOS** | -0.109 | 0.1032 | (0.097, 0.481) | | 0.289 |
|  | **Normal** | Reference |  |  | |  |
|  | **Age** | 0.007 | 0.0034 | (0.001, 0.014) | | **0.030** |
|  | **BMI** | 0.002 | 0.0052 | (-0.008, 0.012) | | 0.697 |
|  | **Baseline TG** | 0.627 | 0.0478 | (0.534, 0.721) | | **<0.001** |
|  | **Time** | 0.000 | 0.0052 | (-0.010, 0.011) | | 0.947 |
|  | **PCOS*Time** | 0.022 | 0.0205 | (-0.018, 0.063) | | 0.274 |
|  | **Normal*Time** | Reference |  |  | |  |
| **TC/HDL-c** |  |  |  |  | |  |
|  | **PCOS** | -0.033 | 0.1744 | (-0.375, 0.309) | | 0.851 |
|  | **Normal** | Reference |  |  | |  |
|  | **Age** | 0.016 | 0.0046 | (0.007, 0.025) | | **<0.001** |
|  | **BMI** | -0.001 | 0.0102 | (-0.021, 0.019) | | 0.945 |
|  | **Baseline TC/HDL-c** | 0.524 | 0.0721 | (0.383, 0.666) | | **<0.001** |
|  | **Time** | -0.108 | 0.0089 | (-0.125, -0.090) | | **<0.001** |
|  | **PCOS*Time** | -0.006 | 0.0233 | (-0.052, 0.040) | | 0.794 |
|  | **Normal*Time** | Reference |  |  | |  |
| **TG/HDL-c** |  |  |  |  | |  |
|  | **PCOS** | -0.124 | 0.1371 | (-0.393, 0.144) | | 0.364 |
|  | **Normal** | Reference |  |  | |  |
|  | **Age** | 0.004 | 0.0042 | (-0.004, 0.013) | | 0.291 |
|  | **BMI** | 0.008 | 0.0061 | (-0.004, 0.020) | | 0.203 |
|  | **Baseline TG/HDL-c** | 0.616 | 0.0334 | (0.551, 0.682) | | **<0.001** |
|  | **Time** | -0.040 | 0.0069 | (-0.053, -0.026) | | **<0.001** |
|  | **PCOS*Time** | 0.023 | 0.0258 | (-0.028, 0.073) | | 0.377 |
|  | **Normal*Time** | Reference |  |  | |  |
| **FPG** |  |  |  |  | |  |
|  | **PCOS** | 0.104 | 0.0995 | (-0.574, 0.902) | | 0.294 |
|  | **Normal** | Reference |  |  | |  |
|  | **Age** | 0.016 | 0.0037 | (0.009, 0.023) | | **<0.001** |
|  | **BMI** | 0.008 | 0.0100 | (-0.011, 0.028) | | 0.413 |
|  | **Baseline FPG** | 0.777 | 0.0884 | (0.604, 0.951) | | **<0.001** |
|  | **Time** | 0.058 | 0.0075 | (0.044, 0.073) | | **<0.001** |
|  | **PCOS*Time** | -0.014 | 0.0140 | (-0.041, 0.014) | | 0.322 |
|  | **Normal*Time** | Reference |  |  | |  |
| **2-h PG** |  |  |  |  | |  |
|  | **PCOS** | 0.221 | 0.3687 | (-0.502, 0.943) | | 0.550 |
|  | **Normal** | Reference |  |  | |  |
|  | **Age** | 0.012 | 0.0112 | (-0.009, 0.034) | | 0.267 |
|  | **BMI** | 0.011 | 0.0195 | (-0.028, 0.049) | | 0.588 |
|  | **Baseline 2-h PG** | 0.659 | 0.0771 | (0.508, 0.810) | | **<0.001** |
|  | **Time** | 0.023 | 0.0154 | (-0.007, 0.054) | | 0.129 |
|  | **PCOS*Time** | -0.009 | 0.0459 | (-0.099, 0.081) | | 0.848 |
|  | **Normal*Time** | Reference |  |  | |  |
| **SBP** |  |  |  |  | |  |
|  | **PCOS** | -2.339 | 1.6648 | (-5.602, 0.924) | | 0.160 |
|  | **Normal** | Reference |  |  | |  |
|  | **Age** | 0.381 | 0.0498 | (0.284, 0.479) | | **<0.001** |
|  | **BMI** | 0.042 | 0.0919 | (-0.139, 0.222) | | 0.651 |
|  | **Baseline SBP** | 0.636 | 0.0446 | (0.549, 0.723) | | **<0.001** |
|  | **Time** | 0.378 | 0.0959 | (0.190, 0.566) | | **<0.001** |
|  | **PCOS*Time** | 0.182 | 0.2562 | (-0.320, 0.684) | | 0.477 |
|  | **Normal*Time** | Reference |  |  | |  |
| **DBP** |  |  |  |  | |  |
|  | **PCOS** | -1.997 | 1.4772 | (-4.892, 0.898) | | 0.176 |
|  | **Normal** | Reference |  |  | |  |
|  | **Age** | 0.190 | 0.0345 | (0.122, 0.257) | | **<0.001** |
|  | **BMI** | 0.251 | 0.0723 | (0.109, 0.393) | | **0.001** |
|  | **Baseline DBP** | 0.473 | 0.0366 | (0.401, 0.545) | | **<0.001** |
|  | **Time** | 0.480 | 0.0771 | (0.329, 0.631) | | **<0.001** |
|  | **PCOS*Time** | 0.123 | 0.2231 | (-0.315, 0.560) | | 0.582 |
|  | **Normal*Time** | Reference |  |  | |  |
| GEE, generalized estimating equation  PCOS, polycystic ovary syndrome, based on NIH criteria  BMI, body mass index; WC, waist circumference; TC, total cholesterol; LDL-c, low density lipoprotein cholesterol;  HDL-c, high density lipoprotein cholesterol; TG, triglycerides; FPG, fasting plasma glucose; 2-h PG, 2-hour plasma glucose; SBP, systolic blood pressure; DBP, diastolic blood pressure  * Indicates interaction. | | | | | | |
